# Liquid Biopsy: A New Avenue for the Diagnosis of Kidney Disease: Diabetic Kidney Disease, Renal Cancer, and IgA Nephropathy

**DOI:** 10.3390/genes15010078

**Published:** 2024-01-07

**Authors:** Jill Dybiec, Weronika Frąk, Joanna Kućmierz, Julita Tokarek, Armanda Wojtasińska, Ewelina Młynarska, Jacek Rysz, Beata Franczyk

**Affiliations:** 1 Department of Nephrocardiology, Medical University of Lodz, ul. Zeromskiego 113, 90-549 Lodz, Poland; 2 Department of Nephrology, Hypertension and Family Medicine, Medical University of Lodz, ul. Zeromskiego 113, 90-549 Lodz, Poland

**Keywords:** liquid biopsy, diabetic kidney disease, renal cancer, nephopathy IgA, chronic kidney disease, microRNA, lncRNAs, piRNAs

## Abstract

Kidney diseases are some of the most common healthcare problems. As the population of elderly individuals with concurrent health conditions continues to rise, there will be a heightened occurrence of these diseases. Due to the renal condition being one of the longevity predictors, early diagnosis of kidney dysfunction plays a crucial role. Currently, prevalent diagnostic tools include laboratory tests and kidney tissue biopsies. New technologies, particularly liquid biopsy and new detection biomarkers, hold promise for diagnosing kidney disorders. The aim of this review is to present modern diagnostic methods for kidney diseases. The paper focuses on the advances in diagnosing three common renal disorders: diabetic kidney disease, renal cancer, and immunoglobulin A nephropathy. We highlight the significance of liquid biopsy and epigenetic changes, such as DNA methylation, microRNA, piRNAs, and lncRNAs expression, or single-cell transcriptome sequencing in the assessment of kidney diseases. This review underscores the importance of early diagnosis for the effective management of kidney diseases and investigates liquid biopsy as a promising approach.

## 1. Introduction

The kidney is a vital organ in the human body responsible for filtering waste products and excess fluids from the blood, producing urine as a result. Located on either side of the spine in the retroperitoneum, kidneys also play a crucial role in regulating electrolyte levels, blood pressure, and the production of red blood cells [1,2]. Each kidney contains around one million nephrons, the functional units responsible for filtration [3]. Each nephron includes a renal corpuscle and a renal tubule, with segments such as the proximal convoluted tubule, loop of Henle, distal convoluted tubule, and connecting tubule. The flow of plasma is filtered at 120 mL/min and undergoes modification in successive nephron segments through the reabsorption of water and essential elements. The distal nephron further adapts the urine composition to the body’s needs [4,5].

Chronic kidney disease is a progressive condition that affects more than 10% of the general population worldwide [6]. Early detection plays a critical role in preventing the onset of kidney failure. Physicians assess patients for indicators of renal disease and perform a range of diagnostic tests to evaluate kidney function, including measurements such as serum creatinine level, cystatin C, blood urea nitrogen, glomerular filtration rate (eGFR), electrolyte levels, and red blood cell count, as well as urinalysis. On occasion, a biopsy of kidney tissue may be required for accurate diagnosis [7,8,9]. To date, these tests have represented the standards for the diagnostics of kidney diseases. However, new technologies offer promising new ways to diagnose and prognose kidney diseases [10]. Biomarkers other than plasma creatinine and the glomerular filtration rate are becoming crucial for the diagnosis and assessment of a patient’s condition. In recent years, updated technical means in liquid biopsy have made it available for wide application in the clinical diagnosis of a range of diseases. Novel research presents an AI-based method using the support vector machine (SVM) algorithm to accurately identify the specific cause of chronic kidney disease through urine peptide analysis. A new visualization and dimensionality reduction algorithms were added to improve the classification performance and help visualize data in 3D space. However, when using uniform manifold approximation and projection (UMAP)-SVM for multiclass classification, it performed less well in differentiating diabetic kidney disease, due to overlapping treatment options with IgAN. Dimensionality reduction techniques such as UMAP can reduce these problems by reducing input factors and improving model performance [11]. Current tools for the diagnosis and monitoring of primary kidney disease, including liquid biopsy biotechniques, will be investigated, and their potential to improve patient conditions will be discussed. Renal diseases include early and chronic allograft rejection, such as donor-derived DNA, exosome, and messenger RNA/ribosomal RNA. The move requires investigation and standard operating procedures. The establishment of a herd and aquatic life at The Cancer Consortium may help expedite the research process, using large-scale research to establish and improve standard procedures [12].

The purpose of this paper is to present new markers in kidney diseases, such as diabetic kidney disease (DKD), kidney cancer, and immunoglobulin A nephropathy (IgAN). We summarized the advances in the diagnosis of renal diseases, in particular the use of liquid biopsy. First of all, we described circulating deoxyribonucleic acid/ribonucleic acid (DNA/RNA), extracellular vesicles, and exosomes, which have promising roles in the management of DKD. We additionally described potential liquid biopsy biomarkers for renal cancer (RC), such as DNA methylation, expression of microRNAs (miRNAs), lncRNAs (long, noncoding RNAs), and P-element-induced wimpy testis (PIWI)-interacting RNAs (piRNAs). Furthermore, we presented the usage of single-cell transcriptome sequencing (scRNA-seq) technology in the diagnosis of IgAN. Therefore, detection of miRNA–messenger RNA (mRNA) networks could potentially be exploited for the development of novel targeted therapies and as biomarkers of IgAN. However, further investigation is crucial to improve our knowledge and, more importantly, accelerate basic research to improve our understanding of the diagnosis and prognosis of kidney diseases.

## 2. Diabetic Kidney Disease

DKD is functional and structural damage to the kidneys that develops as a result of chronic hyperglycemia. It is a serious complication of type 1 diabetes and type 2 diabetes [13]. The kidney’s filtering system becomes increasingly damaged over the years. Early treatment can prevent or slow down this disease and reduce the risk of complications. As diabetic kidney disease progresses, it can lead to end-stage kidney disease, which is a life-threatening condition [14]. Treatment options for such kidney failure include dialysis or kidney transplant. A narrower term is “diabetic glomerulopathy”, which is diagnosed after performing a kidney biopsy and detecting changes typical of DKD in the glomeruli. Some patients with type 2 diabetes have chronic kidney disease (CKD) that does not develop as a result of diabetes or for which diabetes is a co-occurring cause [15]. Recently, new options have been explored to replace renal tissue biopsy to confirm DKD. This biopsy is quite invasive and may result in damage to the tissue surrounding the diseased kidney tissue. It is worth focusing primarily on liquid biopsy based on circulating DNA/RNA, extracellular vesicles, and exosomes. 

Exosomes may play crucial roles in the interaction between kidney cells and the progression of diabetic nephropathy. In order to investigate the relationship and interaction between proximal tubular epithelial cells (PTEC) and mesangial cells (MC), a study was conducted, which included healthy individuals and patients with type 2 diabetes. The study results showed that exosomal miR-92a-1 derived from PTEC 5p modulated the renal microenvironment in in vivo and in vitro models, resulting in the progression of diabetic nephropathy. Furthermore, the blocking of the epigenetic regulatory network of miR-92a-1-5p offers hope for the use of a new therapeutic strategy [16]. Exosomes, produced by cells in different parts of the nephron, can carry protein markers, mRNA, and microRNA (miRNA) related to abnormal kidney function or structural damage. Urinary exosomes offer a comprehensive perspective on the entire urinary system [17]. Previous studies have indicated its potential involvement in the pathogenesis of diabetic nephropathy, including the control of fibrosis through transforming growth factor β1, the extracellular matrix and cell adhesion proteins, cellular hypertrophy, growth factor regulation, cytokine production, and activation of the redox system [18]. 

Exosomes can function as paracrine effectors and mediate intercellular communication. They are stable in biological fluids and paraffin-embedded sections, making them suitable as “liquid biopsy” or disease-specific biomarkers. Several studies have investigated the correlation between urinary microRNAs and blood glucose levels, demonstrating that the concentrations of various extravesicular miRNAs in urine (such as miRNA-941-5p, miRNA 34c-5p, and miRNA-208a-3p) are associated with glycosylated hemoglobin levels. Urine microRNAs have become a new, non-invasive liquid biopsy for disease diagnosis [19,20]. Recent research shows that urine proteomics can be used as a non-invasive method to diagnose DKD and identify high-risk patients to monitor disease progression [21]. Evidence also indicates that the early detection of diabetic nephropathy may be possible through analyzing NGAL and SMAD1 gene expression in peripheral blood and urine samples [22].

The increasing significance of miRNAs as biomarkers in identifying and forecasting the advancement of kidney diseases should also be highlighted. New approaches are being created to identify miRNAs in fluid biopsies, such as urine, plasma, serum, and other bodily fluids. Since they act as functional transcripts that control the expressions of numerous protein-coding genes, disparities in miRNA patterns during disease offer important insights into the fundamental disease mechanisms and pathways [23].

D-serine, found in minimal levels only in humans, serves as a biomarker for kidney diseases and can pinpoint the source of such conditions. Typically, diagnosing these requires a kidney biopsy. Studies have revealed that diabetic nephropathy leads to a higher fractional excretion of d-serine. Profiling d-serine in patients with kidney diseases aids in guiding proper treatment by assisting in the diagnoses of the origins of these conditions. These findings highlight the potential of d-serine as a non-invasive biomarker for kidney diseases, offering a promising alternative to invasive procedures like kidney biopsies for diagnosis [24].

## 3. Renal Cancer

**Figure 1:**
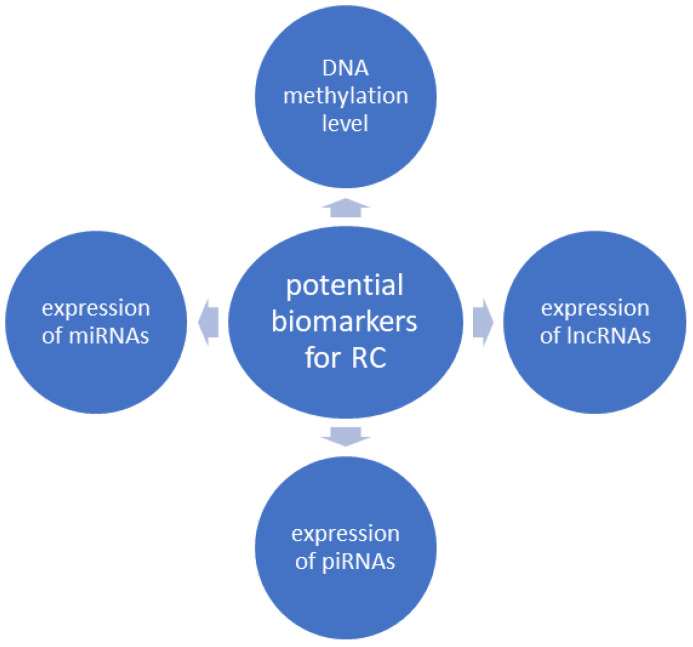
Most important potential liquid biopsy biomarkers for renal cancer. miRNAs—microRNAs, lncRNAs—long noncoding RNAs, piRNAs—P-element-induced wimpy testis-interacting RNAs, RC—renal cancer.

Renal cancer accounts for approximately 3% of all cancers worldwide, and its incidence increases every year [25]. Renal cell carcinoma (RCC) is the most common histologic subtype of RC and, moreover, the most lethal cancer of the urinary system [26,27]. Even though RCC survival rates have significantly improved recently, up to one-third of patients already have regional or distant metastases at the time of diagnosis, which reduces their 5-year overall survival rate [28,29]. Therefore, this highlights the need for effective screening methods and the development of novel diagnostic tools that will enable the early detection of RC [29,30]. Currently available diagnostic strategies for RC include contrast-enhanced computed tomography (CECT) and ultrasonography (USG). However, CECT is considered an invasive procedure with high irradiation and excessive costs, and the accuracy of the ultrasonographic screening depends on the skills of the examiner, as well as the size and location of the tumor [29]. Some of the serum and urine biomarkers have been taken into consideration as potential screening tools but have not achieved validation yet [31]. Liquid biopsy involves detecting epigenetic changes in the nucleic acids present in body fluids (usually blood) [10]. Many epigenetic changes, such as DNA methylation, expression of miRNAs, and lncRNAs, occur in the cells during the process of carcinogenesis. These biomarkers can easily be found in urine, serum, or plasma and function as early cancer detectors. The presence of tumor-derived nucleic acids might be detected in biofluids even before morphological disturbances can be noticed using conventional examination methods, making the liquid biopsy a promising way of screening in the future [32]. Furthermore, the minimally invasive form is an undeniable advantage of this screening strategy [10]. The most important potential liquid biopsy biomarkers for RC are presented in Figure 1.

### 3.1. DNA Methylation

DNA methylation is a process in which a methyl group (-CH_3_) is added at the carbon-5 position of cytosine residues in CpG dinucleotides via the action of DNA methyltransferase (DNMTs) enzymes, resulting in 5-methylcytosine (5mC) formation. This common epigenetic mechanism occurs in both normal and pathological biological processes and is responsible for regulating gene expression [33]. Promoter CpGs mostly remain un- or hypomethylated in normal cells, allowing the active expressions of the genes. On the other hand, hypermethylation of the promoter CpGs in tumor suppressor genes could lead to the inhibition of this anti-oncogenic pathway and might appear in different cancer types, including RC [34]. Changes in DNA methylation are considered early markers of carcinogenesis; they might be observed even in the precancerous stage of RCC [32,35].

The most widely studied genes, considering their methylation, include VHL, APC, RASSF1A, P16, P14, RARB, TIMP3, and GSTP1 [32]. However, studies have found only a few biomarkers and multi-marker panels characterized by over 70% sensitivity [32,36,37]. Furthermore, the studies were mostly focused on common tumor suppressor genes, which could be methylated in various cancer types and present low specificity for RCC. The most promising method for finding biomarkers in biofluids appears to be next-generation sequencing-based biomarker hunting, but larger studies should be conducted to obtain reliable conclusions on this topic [32].

### 3.2. Expression of miRNAs

MiRNAs consist of small, noncoding RNAs that play a vital role in the regulation of target gene expression by binding to the 3′ untranslated region (3′UTR) of mRNA, resulting in its degradation or a suppression of translation [38]. Studies have suggested that miRNAs are involved in the process of carcinogenesis on many different levels, including angiogenesis, apoptosis, metastasis, invasion, and cell proliferation [39,40,41,42]. The abnormal expression of miRNAs has been found, among others, in the pathogenesis of RCC [43]. This type of nucleic acid has many advantages as a potential biomarker; it can be found in various biofluids (urine, serum, plasma, and saliva), it is characterized by the high stability of the molecules, and the normal expression profile does not change depending on the patient’s gender or age [32]. Moreover, many studies have shown that miRNA levels vary depending on the phase of treatment of RCC, which might be used as an efficient monitoring marker during RCC treatment [32,44,45,46,47]. Moreover, specific miRNA levels have been associated with particular clinical parameters in RCC. These dependencies are presented in Table 1.

The most widely evaluated type of circulating miRNA in relation to RCC is miR-210. Studies have shown that the levels of this biomarker are increased in patients with RCC, in comparison to healthy controls. However, increased levels of miR-210 occur in other cancers as well and in other non-malignant conditions. Consequently, further research on this topic is necessary for further validation of this parameter in clinical usage [32,56,57,58].

In summary, miRNAs offer promising possibilities for future usage as both diagnostic and prognostic biomarkers of RCC; however, standardized research is needed to achieve more reliable results [32].

### 3.3. Expression of lncRNAs

lncRNAs is a group of RNAs with over 200 nucleotides in its length that is involved in the regulation of gene expression via the modification of chromatin or post-transcriptional interactions with miRNAs, mRNAs, and proteins [59,60]. Studies have found that lncRNAs influence the development of many cancerous diseases, including RC. These molecules could interfere with many processes during carcinogenesis, such as angiogenesis, apoptosis, metastasis, invasion, cell proliferation, and drug resistance [32,61].

Similar to miRNAs, lncRNAs are characterized by high stability and are protected from degradation in body fluids. These nucleic acids are also more specific than other RNA types [62]. As a result, lncRNAs might offer more accurate diagnostic and prognostic information and are of great interest as potentially highly specific non-invasive biomarkers.

However, studies investigating lncRNAs mostly used a small number of samples and analyzed only one type of this nucleic acid [32]. Thus, more complex research is mandatory to fully analyze this group as potential biomarkers. Due to their high specificity, the hopes for lncRNAs to become a future biomarker of RCC are relatively high.

### 3.4. Other Epigenetic Changes

Other epigenetic changes that could be involved in new screening methods include circulating nucleosomes and their alterations and other noncoding RNAs, e.g., piRNAs [63,64]. Studies have suggested that piRNAs may be involved in the development of cancer by inhibiting or degrading tumor suppressor genes or oncogenes. Moreover, RCC has been associated with the dysregulation of several piRNAs [65]. Although piRNAs might become a new fluid biomarker of RCC, further studies on this topic are necessary to fully assess its potential [32].

## 4. Nephropathy IgA

Immunoglobulin A nephropathy is the disease that is the most common primary glomerulonephritis worldwide [66], and the prevalence of IgAN is f 2.5 per 100,000 per year [67]. The beginning of this condition falls in the second and third decades, with a 2:1 male-to-female ratio [67]. Epidemiological data suggest that 40% of patients, within 10 to 20 years from the moment of diagnosis, will suffer from chronic kidney disease, including end-stage kidney disease (ESKD) [68,69]. Clinical symptoms like proteinuria, hypertension, hypoproteinemia, hyperuricemia, and impaired renal function are main predictors of a poor prognosis for IgAN [69]. The etiology of IgAN is unknown; however, the role of dysregulated T-cells and their immune response to viral, bacterial, and food antigens, which lead to the activation of mucosal plasma cells and the production of polymeric IgA, is emphasized [70]. Diagnosis of IgAN is possible with a kidney biopsy [66]. In the immunofluorescence microscopy, there are dominant or codominant IgA mesangial stainings with lesser extents of IgG and IgM. C3 is present in more than 90% of the biopsies with IgAN, which suggests an activation of the alternative complement pathway [71]. In the electron microscopy, we could observe mesangial and, occasionally, subendothelial deposits of IgA [72]. There are new findings in studies embracing IgAN, which could enhance both the diagnosis and its treatment.

### 4.1. Usage of Single-Cell Transcriptome Sequencing (scRNA-seq) Technology in the Diagnosis of IgAN

Single-cell transcriptome sequencing technology was discovered in 2009 and has become the advanced way of detecting the heterogeneity and complexity of RNA transcripts, not only within individual cells but also in the composition of different cell types and functions within highly organized tissues [73]. The mentioned technology was used by Zheng et al. [74]. They performed scRNA-seq in kidneys and CD14^+^ peripheral blood mononuclear cells of thirteen IgAN patients and six controls, which revealed high expressions of the pathological genes in the mesangium, epithelium, and immune cells. The joining chain of multimeric IgA and IgM (JCHAIN) gene was found to be upregulated in the mesangial cells, which could pose a potential correlation with the deposition of the IgA. Moreover, there was an overexpression of genes in the mesangium, which is responsible for encoding collagens, glycoproteins, and integrins. They also found high levels of genes correlated with inflammation, such as cytokines and chemokines, and observed an increased cell-type-specific interaction gene expression between the glomerulus and the tubule-interstitium.

### 4.2. Identification of the miRNA–mRNA Network in IgA Nephropathy 

MiRNAs are small, non-coding endogenous RNAs that are made of 22 nucleotides, and their essential role is to influence mRNA through recognition sites in the UTR to regulate their stability [75]. MiRNAs regulate RNA silencing and gene expression and participate in various cellular processes, thus influencing cell homeostasis [76]. Research on the involvement of miRNAs in the development of kidney disease remains not fully studied but is rapidly expanding. Several studies revealed that miRNAs and the expression levels of their downstream target genes are potentially important in the diagnosis and pathogenesis of IgAN [77].

Wei et al. identified 4 downregulated and 16 upregulated DE-miRNAs in the urine sediments of IgAN patients and normal controls. It is worth emphasizing that detection of urine is easy, non-invasive, and inexpensive [75]. Those features are extremely important because a biopsy may not be performed, due to some physical factors, such as hypertension, anatomical variation, and pregnancy, and because of its non-invasive detection of IgA nephropathy, it has become an urgent issue [78]. Moreover, urine is an optimal material, due to the correlation between intrarenal and urinary miRNA levels [79]. The summary of the most significant miRNA types is shown in Table 2.

The presented studies showed that miRNA–mRNA regulatory axes play important roles in the pathogenesis and diagnosis of IgA. There is a need for further verification of the miRNA–mRNA network in future experiments, which may assist in IgAN treatments by targeting established miRNA–mRNA interaction networks [84]. 

### 4.3. Pathogenic Importance of Aberrantly Glycosylated IgA1 in IgAN

As mentioned above, levels of the polymeric form of IgA1 are elevated in the serum of patients suffering from IgAN [85,86]. The process of producing abnormal IgA, and therefore kidney damage, begins with the formation of a deficiency of galactose O-glycans in the hinge region of IgA1. This galactose-free IgA1 (Gd-IgA1) is composed of terminal N-acetylgalactosamine (GalNAc) or sialylated GalNAc [87,88]. Compared to the serum of the healthy population, IgA1 contains little or no galactose-deficient O-glycans [89]. Recent studies have shown that enzymatic processes of creating Gd-IgA1 and GalNAc may be regulated by genetic mechanisms [90], and many of them are induced by interleukin (IL)-6 and IL-4 [90]. The concentration of immune complexes made of Gd-IgA1 is increased in the blood and urine of patients with IgAN; thus, increased serum Gd-IgA1 levels have a pivotal role in the pathogenesis of IgAN and are associated with the exacerbation of proteinuria and a greater risk of deterioration of renal function in IgAN [91]. Suzuki et al. reported that serum levels of IgA, Gd-IgA1, Gd-IgA1-specific IgG (the best selected parameter with a sensitivity of 89% and specificity of 92%), and Gd-IgA1-specific IgA were elevated in patients with IgAN, compared to both the group of healthy patients and patients with other renal diseases [92]. Importantly, they indicated that a set of mentioned serum biomarkers may be helpful to differentiate IgAN from other glomerular diseases. Moreover, they devised a novel lectin-independent method embracing a monoclonal antibody (KM55 mAb) for measuring the level of Gd-IgA1 in patients’ serum, which could be used worldwide for the standardized measurement of serum Gd-IgA1 [93]. 

## 5. Conclusions

Renal physiology is associated with essential metabolic procedures, such as removing waste products from the blood and regulating the levels of compounds, like hormones, water–electrolyte balance, and the maintenance of blood pressure. Prolonged oxidative stress; comorbidities, e.g., diabetes mellitus or heart diseases; or isolated renal disorders, such as renal cancer and IgA nephropathy, lead to disturbances in kidney regular function and the homeostasis of the whole body. Given the above, early detection of kidney dysfunction is crucial to survival. This review highlighted modern diagnostic approaches, with specific focuses on diabetic kidney disease, renal cancer, and immunoglobulin A nephropathy. Liquid biopsy, specifically examining exosomes and miRNAs, emerges as a less invasive alternative to traditional biopsies for DKD diagnosis. Exosomes carrying various markers related to abnormal kidney function offer insights into diabetic nephropathy’s pathogenesis. The research of exosomal miR-92a-1 as a modulator of the renal microenvironment presents a novel therapeutic avenue, emphasizing the potential of liquid biopsy in shaping treatment strategies. Renal cancer, constituting 3% of global cancers, demands early detection for improved survival rates. Current diagnostic methods, such as contrast-enhanced computed tomography and ultrasonography, have limitations, prompting the exploration of liquid biopsy. Epigenetic changes, including DNA methylation and miRNA expression, are potential biomarkers for RC. We emphasize the need for standardized research to establish the reliability of these markers. Immunoglobulin A nephropathy, the most common primary glomerulonephritis, often leads to chronic kidney disease. The traditional diagnostic approach involves kidney biopsy, but advancements like single-cell transcriptome sequencing (scRNA-seq) and miRNA analysis in urine sediments provide new perspectives. The scRNA-seq reveals gene expression patterns in IgAN patients, while miRNA–mRNA regulation emerges as an important player in the disease’s pathogenesis and diagnosis. In conclusion, the integration of liquid biopsy into diagnostic strategies for these kidney conditions represents a revolutionary change. We underscore the potential of liquid biopsy in detecting early-stage diseases and monitoring treatment responses. Our review discussed DKD, RC, and IgAN in terms of new specific liquid biopsy markers, such as exosomes, DNA methylation, and miRNAs, demonstrating the evolving kidney disease diagnostics. Further research is necessary to validate these liquid biopsy biomarkers and establish their clinical utility in routine practice.

## Figures and Tables

**Table 1:** Associations between specific miRNA levels and particular clinical parameters in renal cell carcinoma. (Fuhrman grade has been replaced by the new International Society of Urological Pathology (ISUP) classification).

miRNA Type	Associated Clinical Parameter	Source
miR-378	advanced tumor stage	[48]
miR-144-3p	advanced tumor stage	[49]
miR-210	advanced tumor stage tumor progression/metastasis	[50]
miR-1233	advanced tumor Fuhrman grade tumor progression/metastasis	[50]
miR-22	advanced tumor stage tumor progression/metastasis	[51]
miR-122-5p	advanced tumor Fuhrman grade	[52]
miR-206	advanced tumor stage advanced tumor Fuhrman grade	[52]
miR-15a	advanced tumor Fuhrman grade tumor necrosis	[53]
miR-508-3p	advanced tumor stage advanced tumor Fuhrman grade tumor progression/metastasis	[54]
miR-885-5p	advanced tumor stage advanced tumor Fuhrman grade	[54]
has-miR-328-3p	tumor progression/metastasis	[55]
has-miR1293	tumor progression/metastasis	[46]
has-miR-301-3p	tumor progression/metastasis	[46]

**Table 2:** Associations between specific miRNAs levels and particular clinical/molecular features in IgAN.

miRNA Type	Features in IgAN	Source
miR-146a	correlated with urinary interleukin (IL)-1β, IL-6, and tumor necrosis factor (TNF)-α expression positively correlated with RANTES (regulated upon activation, normal T-cell expressed, and secreted)	[80]
miR-155	inversely correlated with urinary IL-1β and TNF-α expression positively correlated with RANTES (regulated upon activation, normal T-cell expressed, and secreted)	[80]
miR-150	significantly upregulated	[81]
miR-150-5p	associated with tubule-interstitial mononuclear infiltrates, especially in lymphoid nodules	[82]
miR-25-3p	significantly unregulated high specificity (79.4%) and sensitivity (87.1%) for diagnosing IgAN	[83]

## Data Availability

Not applicable.
